# Yellow Fever

**Published:** 1878-10

**Authors:** 


The Bistoury.

ELMIRA, N. Y., OCTOBER, 1878.

The BISTOURY is published Quarterly, upon the 1st of April, July, October and January, at Fifty Cents a year in advance.

### YELLOW FEVER.

Terrible reports are reaching us daily from the fever stricken districts of the South. Since the introuction of yellow fever into this country in 1669, no more devastating attack of the epidemic has occurred than the present one.

A history of the disease in this country, shows that it has appeared in Philadelphia ten times, New York five times, Boston twice, Providence twice, New Orleans twenty-four times, Charleston seven times, Mobile four times.

The fever usually attacks large towns, located upon level, low ground, near a still running river, and first makes its appearance in the uncleanly, badly drained portions of the city, where sewers and gutters are foul and where vegetable matter is decomposing along the river bank. Low, moist or wet places, and want of cleanliness in cellars and yards render the conditions favorable for an attack of the disease. Ships, from bilge water and garbage in the hold, often give rise to the pestilence. In short, wherever filth, imperfect drainage, impure water. decaying vegetable and animal matter abounds, there rollicks the pestilence.

A hot summer, or a high temperature long maintained, ranging from 72° to 90° Fahr. is favorable to its propagation.

Elevated situations, away from the river and city, are apt to be free from it. Hence the advisability of removing from an infected city upon the first appearance of the disease.

Acclimated persons, or those who have always resided in the South, are not so apt to contract the disease as those who visit the infected districts. Negroes are more exempt than the white people, although the present epidemic has proved quite fatal to them. Persons who have once had the disease and recover, never have it again if they continue to remain in the same climate. Should such persons remove to a northern latitude for a season, however, then return, they at once become liable to another attack.

Medical men are not agreed as to the exact cause of yellow fever. The most plausible theory is that the poisonous sporules—whatever they may be, are introduced into the circulation, as in other miasmatic fevers, and destroy the red corpuscles of the blood. The blood so impoverished loses its capability of nourishing the body, and then follows the long train of symptoms that naturally arise from such a condition of affairs.

The period of incubation—the time from the absorption of the poison until the disease manifests itself, is usually from one to three days. The first symptons complained of are headache, weakness, loss of appetite and pain in the limbs. Some times it comes on with a chill, strong men being suddenly stricken down while at work and become immediately alarmingly ill, complaining of severe pain in the head and limbs, while the face is hot and the eyes possessed of a peculiar lustre. Twelve hours from the beginning of the attack an offensive smell is emitted from the body of the patient, the tongue becomes covered with a yellowish fur, and the gums appear red and swollen, and bleed upon being touched. Drink and food are at once rejected by the stomach. Vomiting becoming frequent and distressing. The bowels are usually constipated and the urine scanty. So the symptoms continue for three days, when the yellow color of eyes and face appear. In the worst cases bleeding from the nose and stomach now set in, which is always regarded as a fatal symptom. In what is termed the third stage of the disease, commencing after the third day, the patient falls into a state of collapse, and is wholly indifferent and helpless to what is transpiring about him. Bleeding from the stomach gives rise to the "black vomit," which is simply the throwing off of the blood that has accumulated there. A deep stupor now comes on, from which the patient does not awake, and death ends the scene.

At this writing, (Sept. 25th) there seems to be no abatement of the dreadful pestilence; no telling when it may subside. If a lower temperature alone is to banish it, another month at least, of suffering and death may ensue, for the disease seems to be beyond the control of the physicians.

At this safe distance, we can but condole with our brethren at the south, send them aid in money, food and medicines, all of which we do right heartily.

It would seem to be a wise course for the National Government to pursue, if a medical commission were sent to the infected districts in the South with a view of learning the causes of the epidemic and discovering, if possible, the means of prevention or cure.

Investigation of this character developed a preventive for small pox, and intermittent fever, and it may be within the range of medical skill to find a means of relief from yellow fever. Our present knowledge of the disease is so meagre as to leave us entirely at the mercy of the scourge.

